# Lung cancer in never-smoker Asian females is driven by oncogenic mutations, most often involving *EGFR*

**DOI:** 10.18632/oncotarget.2925

**Published:** 2015-02-28

**Authors:** Sang Yun Ha, So-Jung Choi, Jong Ho Cho, Hye Joo Choi, Jinseon Lee, Kyungsoo Jung, Darry Irwin, Xiao Liu, Maruja E. Lira, Mao Mao, Hong Kwan Kim, Yong Soo Choi, Young Mog Shim, Woong Yang Park, Yoon-La Choi, Jhingook Kim

**Affiliations:** ^1^ Department of Pathology and Translational Genomics, Samsung Medical Center, Sungkyunkwan University School of Medicine, Seoul, Korea; ^2^ Samsung Biomedical Research Institute, Samsung Medical Center, Sungkyunkwan University School of Medicine, Seoul, Korea; ^3^ Department of Thoracic and Cardiovascular Surgery, Samsung Medical Center, Samsung Biomedical Research Institute, Sungkyunkwan University School of Medicine, Seoul, Korea; ^4^ Samsung Advanced Institute for Health Sciences & Technology, Sungkyunkwan University School of Medicine, Seoul, Korea; ^5^ Agena Bioscience, Sequenom, San Diego, CA, USA; ^6^ BGI-Shenzhen, Shenzhen, China; ^7^ Department of Biology, University of Copenhagen, Copenhagen, Denmark; ^8^ Oncoloy Research Unit, Pfizer Worldwide Research and Development, San Diego, CA, USA; ^9^ WuXi AppTec, Shanghai, China; ^10^ Samsung Genome Institute, Samsung Medical Center, Sungkyunkwan University School of Medicine, Seoul, Korea

**Keywords:** non-small cell lung cancer, adenocarcinoma, never-smoker female, driver mutation, EGFR

## Abstract

The aim of this study was to determine the distribution of known oncogenic driver mutations in female never-smoker Asian patients with lung adenocarcinoma. We analyzed 214 mutations across 26 lung cancer-associated genes and three fusion genes using the MassARRAY® LungCarta Panel and the *ALK, ROS1*, and *RET* fusion assays in 198 consecutively resected lung adenocarcinomas from never-smoker females at a single institution. *EGFR* mutation, which was the most frequent driver gene mutation, was detected in 124 (63%) cases. Mutation of *ALK, KRAS, PIK3CA, ERBB2, BRAF, ROS1,* and *RET* genesoccurred in *7%*, 4%, 2.5%, 1.5%, 1%, 1%, and 1% of cases, respectively. Thus, 79% of lung adenocarcinomas from never-smoker females harbored well-known oncogenic mutations. Mucinous adenocarcinomas tended to have a lower frequency of known driver gene mutations than other histologic subtypes. *EGFR* mutation was associated with older age and a predominantly acinar pattern, while *ALK* rearrangement was associated with younger age and a predominantly solid pattern. Lung cancer in never-smoker Asian females is a distinct entity, with the majority of these cancers developing from oncogenic mutations.

## INTRODUCTION

Lung cancer is the leading cause of cancer-related mortality, with 1.38 million annual deaths worldwide [1]. Tobacco smoking is the main risk factor for lung cancer; however, approximately 25% of lung cancers worldwide occur in never-smokers [2, 3]. Moreover, the risk of lung cancer differs by race/ethnicity. In the United States, approximately 10% of patients with lung cancer are never-smokers [4], while in Asia, >30% of patients with lung cancer are never-smokers and ≥50% of lung cancers occur in women who are never-smokers [5]. Never-smoker East Asian females have a tendency to develop adenocarcinoma, and these never-smokers exhibit higher treatment response rates to epidermal growth factor receptor tyrosine kinase inhibitors (EGFR-TKIs), such as gefitinib and erlotinib, than those with a history of tobacco smoking [6]. In several phase III studies, significantly better response rates and longer progression-free survival were observed in advanced non-small cell lung cancer (NSCLC) patients harboring activating *EGFR* mutations who were treated with first-line EGFR-TKIs than those patients treated with doublet platinum-based chemotherapy [7, 8]. In addition, a fusion protein of the N-terminal portion of the *echinoderm microtubule-associated protein-like 4* (*EML4*) gene and the intracellular signaling portion of the *anaplastic lymphoma kinase* (*ALK*) tyrosine kinase receptor has been identified in a small subset of NSCLC patients [9]. Patients harboring the *EML4-ALK* fusion show unique clinicopathologic and physiological characteristics and respond positively to *ALK* inhibitors [10–12]. As for *EGFR* mutations, several reports have identified the *EML4-ALK* fusion protein predominantly in young female never-smokers with adenocarcinoma, although the presence of this fusion protein is mutually exclusive with *EGFR* mutation [9, 13]. More specifically, the *EML4-ALK* fusion gene was found in 23.7% of never-smoker female lung adenocarcinoma patients [14].

Lung adenocarcinoma in never-smoker females has been established as a distinct entity based on its particular epidemiologic, clinical, and biological characteristics. *EGFR* mutations and the *EML4-ALK* translocation are defined as driver mutations because these alterations are responsible for both initiation and maintenance of lung cancer. The discovery of driver oncogene genetic variants that are sensitive to molecular-targeted drugs is crucial for improvement of treatment strategies. Therefore, use of multi-mutational profiling in lung cancer studies is important for identification of driver gene alterations in order to validate the effectiveness of molecular-targeted therapies. In addition, the proportion of never-smokers with lung cancer is likely to increase as a result of smoking cessation and prevention programs. Thus, the aims of this study were to analyze the distribution of oncogenic driver mutations and to compare these mutations with clinicopathologic characteristics in female Asian never-smoker lung adenocarcinoma patients.

## RESULTS

### Patient population and histopathologic classification

The patient population is summarized in Table 1. All patients were women with no smoking history. Median age at diagnosis was 60 years (range, 29–81 years). Most patients (85.3%) had undergone lobectomy. Two patients had pleural metastasis at the time of surgery. Eleven (5.6%) patients had received neoadjuvant concurrent chemoradiation therapy. Among the 198 patients in this study, 104 (52.5%) patients had T1 stage tumors, 83 (41.9%) patients had T2 stage tumors, and 11 (5.6%) patients had T3 stage tumors. Nine patients did not undergo lymph node dissection and were excluded from the analysis of N stage. N0, N1, and N2 stage tumors were observed in 124 (65.6%), 25 (13.2%), and 40 (21.2%) patients, respectively. Two of the 198 cases were classified as minimally invasive adenocarcinomas with an invasive component of < 5 mm. Invasive adenocarcinomas were classified as predominantly acinar (142 cases, 71.7%), predominantly papillary (18 cases, 9.1%), predominantly solid (17 cases, 8.6%), predominantly lepidic (9 cases, 4.5%), and predominantly micropapillary (1 case, 0.5%) patterns. Nine (4.5%) cases were classified as mucinous adenocarcinoma. Two minimally invasive adenocarcinomas were classified as predominantly lepidic for statistical analysis. In addition, all histologic patterns that were observed in >10% of the tumor area were recorded. An acinar pattern was observed in 169 (85.4%) cases. Papillary, lepidic, micropapillary, solid, and mucinous patterns were observed in 68 (34.3%), 36 (18.2%), 36 (18.2%), 33 (16.7%), and 17 (8.6%) cases, respectively.

**Table 1:** Patient population

Characteristics	Number of patients (%)
Age, years	median 60 (range, 29–81)
Histologic classification	
Minimally invasive adenocarcinoma	2 (1.0)
Invasive adenocarcinoma	
Lepidic predominant	9 (4.5)
Acinar predominant	142 (71.7)
Papillary predominant	18 (9.1)
Micropapillary predominant	1 (0.5)
Solid predominant	17 (8.6)
Mucinous adenocarcinoma	9 (4.5)
T stage	
1	104 (52.5)
2	83 (41.9)
3	11 (5.6)
N stage^1^	
0	124 (65.6)
1	25 (13.2)
2	40 (21.2)
M stage	
0	196 (99.0)
1	2 (1.0)
Operation	
Wedge resection	16 (8.0)
Lobectomy	169 (85.3)
Lobectomy + α^2^	13 (6.6)
Neoadjuvant CCRT^3^	
Yes	11 (5.6)
No	187 (94.4)

1 Nine cases were excluded because lymph node dissection was not performed.

2 α includes lobectomy with wedge resection of the other lobe, bilobectomy, or pneumonectomy.

3 CCRT indicates concurrent chemoradiation therapy.

### Fusion gene and LungCarta analyses

The results of the fusion gene and LungCarta analyses are summarized in Figure 2 and Table 2, and a full list of mutations that were identified is shown in Supplemental Table 1. Among the 198 cases, driver mutations were detected in 157 (79%) cases. *EGFR* mutations were the most frequently found mutation in lung adenocarcinomas of female never-smokers (124 cases, 63%). *EGFR* mutations were detected in exon 19 in 47 (24%) cases, exon 20 in 16 (8%) cases, exon 21 in 58 (29%) cases, exons 18 and 21 in 2 (1%) cases, and exons 18 and 20 in 1 (1%) case. *ALK* rearrangement was observed in 14 (7%) of cases. The frequencies of *KRAS*, *PIK3CA*, *TP53*, *ERBB2*, and *BRAF* mutations were 4%, 2.5%, 2%, 1.5%, and 1%, respectively. *ROS1* and *RET* gene rearrangements were each found in 2 (1%) cases. *ALK*, *ROS1*, and *RET* gene fusions were mutually exclusive with *EGFR* and *KRAS* mutations. The gene mutations were mutually exclusive with the exception of concurrent mutation of *EGFR*/*PIK3CA* (*n* = 3), *EGFR*/*TP53* (*n* = 1), *ALK*/*TP53* (*n* = 2), *KRAS*/*PIK3CA* (*n* = 1), and *KRAS*/*ERBB2* (*n* = 1; Figure 3). The results of Sanger sequencing validation were consistent with the results of LungCarta analyses in all cases (Supplemental Table 2).

**Figure 1:**
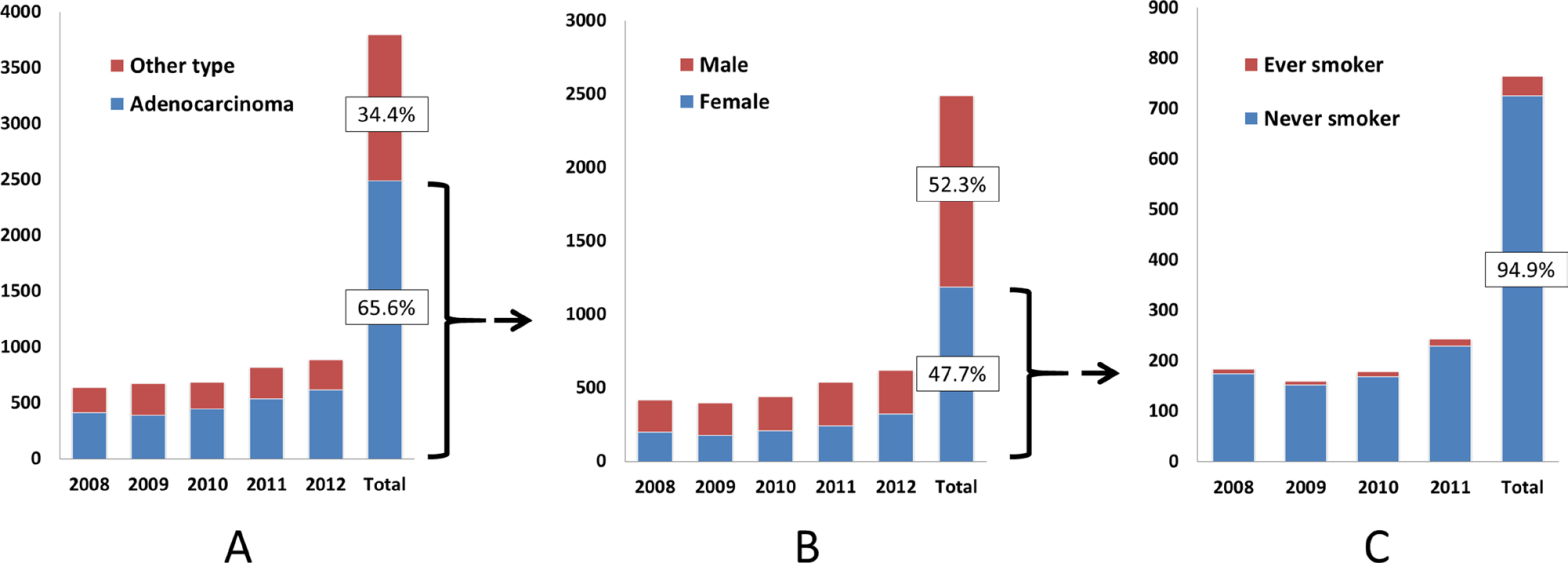
Process of patient (female never smoker with lung adenocarcinoma) selection in this study **(A)** Distribution of lung cancer according to histology subtype. **(B)** Distribution of lung adenocarcinoma according to gender. **(C)** Distribution of lung adenocarcinoma from female according to smoking status. Patients with no medical record of smoking status were excluded. The record of year 2012 was not shown due to lack of smoking information.

**Figure 2:**
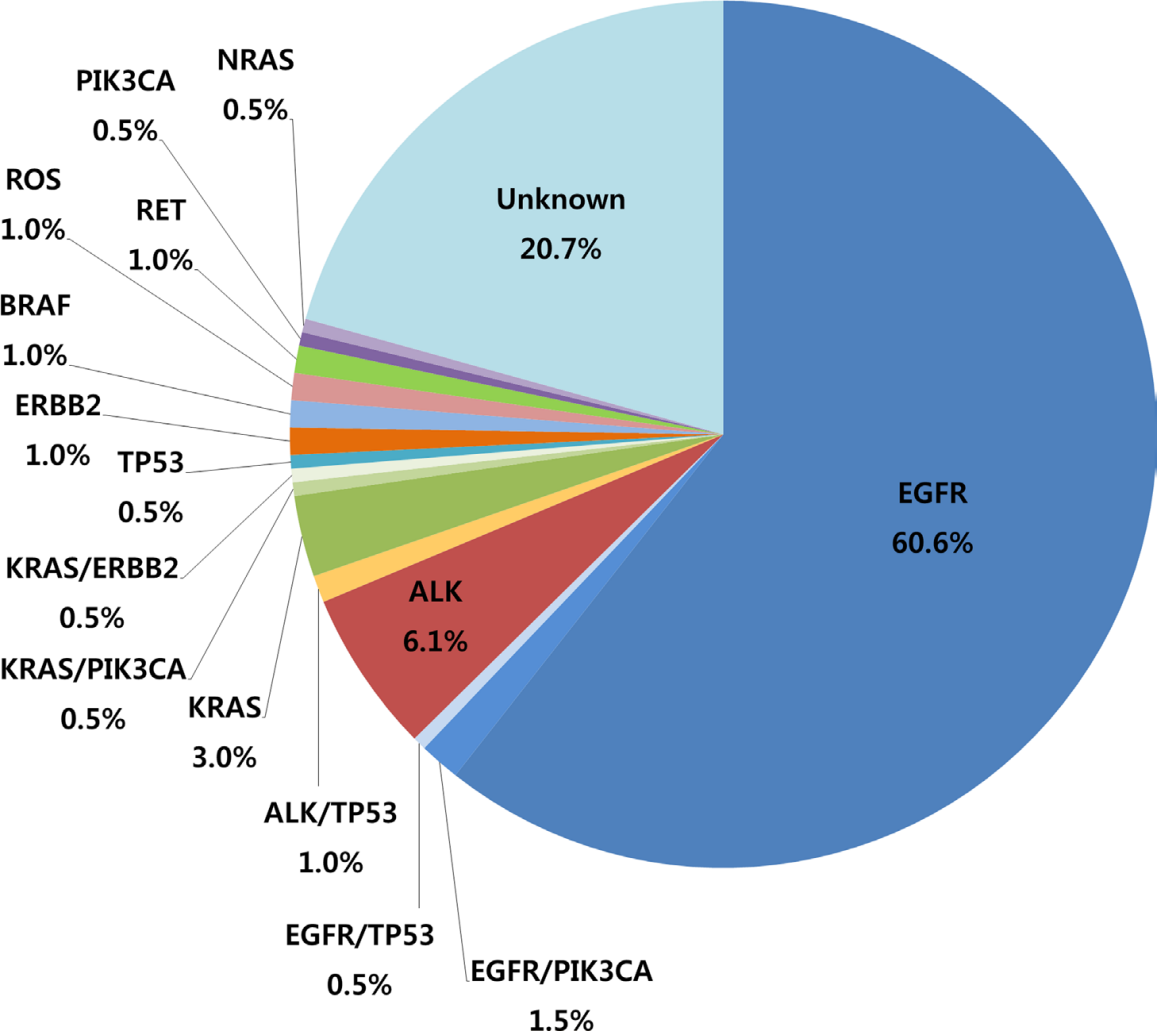
Frequency of driver gene mutations in lung adenocarcinomas from East Asian never-smoker females

**Table 2:** Relationship between driver mutation status and histologic subclassification of adenocarcinoma according to predominant type

	Total	Lepidic^1^	Acinar^2^	Papillary	Micropapillary	Solid	Mucinous^3^	*p*-value
**n (%)**	**198 (100)**	**11 (5.6)**	**142 (71.7)**	**18 (9.1)**	**1 (0.5)**	**17 (8.6)**	**9 (4.5)**	
**Wild-type**	41 (20.7)	3 (7.3)	29 (70.7)	2 (4.9)	0 (0)	3 (7.3)	4 (9.8)	0.446 Mucinous vs. others: 0.090
**Mutant type**	157 (79.3)	8 (5.1)	113 (72.0)	16 (10.2)	1 (0.6)	14 (8.9)	5 (3.2)	
***EGFR***	124 (62.6)	7 (5.6)	102 (82.3)	9 (7.3)	1 (0.8)	5 (4.0)	0 (0)	< 0.001
***ALK***	14 (7.1)	0 (0)	6 (42.9)	2 (14.3)	0 (0)	5 (35.7)	1 (7.1)	0.012
***KRAS***	8 (4.0)	1 (12.5)	1 (12.5)	0 (0)	0 (0)	2 (25.0)	4 (50.0)	< 0.001
***PIK3CA***	5 (2.5)	0 (0)	3 (60.0)	1 (20.0)	0 (0)	0 (0)	1 (20.0)	0.31
***TP53***	4 (2.0)	0 (0)	4 (2.8)	0 (0)	0 (0)	0 (0)	0 (0)	1
***ERBB2***	3 (1.5)	0 (0)	2 (66.7)	0 (0)	0 (0)	0 (0)	1 (33.3)	0.272
***BRAF***	2 (1.0)	0 (0)	0 (0)	2 (100)	0 (0)	0 (0)	0 (0)	0.043
***ROS1***	2 (1.0)	0 (0)	0 (0)	1 (50.0)	0 (0)	1 (50.0)	0 (0)	0.086
***RET***	2 (1.0)	0 (0)	1 (50.0)	1 (50.0)	0 (0)	0 (0)	0 (0)	0.487
***NRAS***	1 (0.5)	0 (0)	0 (0)	0 (0)	0 (0)	1 (100)	0 (0)	0.192

1 Two cases of minimally invasive adenocarcinoma were included in the lepidic predominant type.

2 Six cases with acinar pattern showed concurrent mutation of *EGFR*/*PIK3CA* (*n* = 3), *EGFR*/*TP53* (*n* = 1), and *ALK*/*TP53* (*n* = 2).

3 Two cases of mucinous adenocarcinoma showed concurrent mutation of *KRAS*/*PIK3CA* (*n* = 1) and *KRAS*/*ERBB2* (*n* = 1).

**Figure 3:**
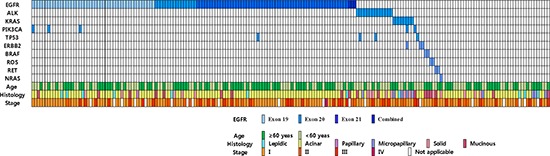
Diagram demonstrating driver gene mutation status and clinicopathologic features in 198 adenocarcinomas from East Asian never-smoker females Most mutations were mutually exclusive with the rare exception of concurrent mutation of *EGFR/PIK3CA* (*n* = 3), *EGFR/TP53* (*n* = 1), *ALK/TP53* (*n* = 2), *KRAS/PIK3CA* (*n* = 1), and *KRAS/ERBB2* (*n* = 1). Patients who received concurrent chemoradiation therapy and those who did not undergo lymph node dissection were excluded in the analysis of TNM stage.

### Relationship between driver mutation status and clinicopathologic characteristics

**Figure 4:**
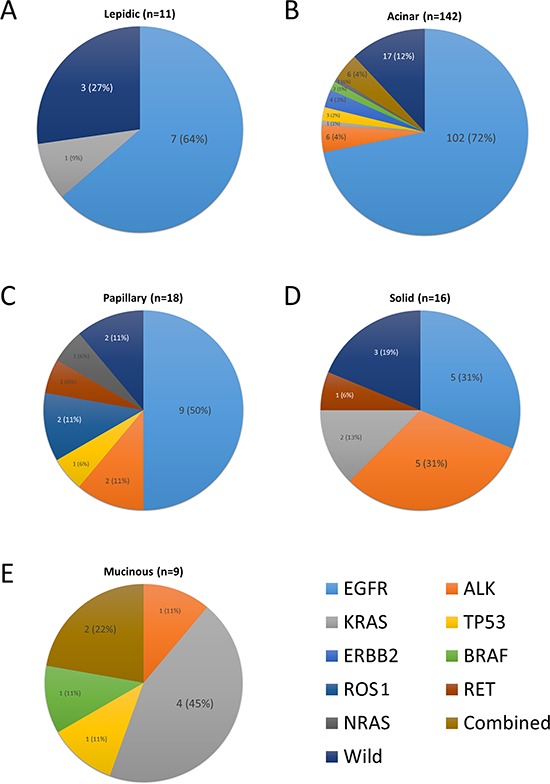
Frequency of driver gene mutations according to predominant histologic subtype **(A)** Lepidic, **(B)** Acinar, **(C)** Papillary, **(D)** Solid, **(E)** Mucinous subtype. In acinar subtype, four cases with concurrent mutations of EGFR/PIK3CA (*n* = 3) and EGFR/TP53 (*n* = 1) are represented as an EGFR mutation, and 2 cases of ALK/TP53 mutation as an ALK mutation. In mucinous subtype, two cases with concurrent mutations of KRAS/PIK3CA and KRAS/ERBB2 are represented as a KRAS mutation.

The relationship between the driver mutation status and histologic subclassification of adenocarcinoma is summarized in Table 2 and Figure 4A–4E. Tumors with *EGFR* mutation exhibited a high frequency of predominantly acinar patterns and an absence of a predominantly mucinous pattern. *ALK* rearrangements were frequently found in cases with a predominantly solid pattern. *KRAS* mutations were detected with high frequency in cases with predominantly mucinous tumors. *BRAF* mutations were detected only in predominantly papillary tumors. These four genes were significantly associated with the histologic subclassification. Mucinous adenocarcinoma tended to harbor a lower frequency of known driver gene mutations than other histologic subtypes (mutation rate, 44.4% vs. 80.4%; *p* = 0.090).

*EGFR* mutations were associated frequently with lepidic and acinar patterns and infrequently with mucinous patterns in cases where the histologic pattern predominated in >10% of the tumor area. *ALK* rearrangement was more frequently detected in tumors with solid and mucinous patterns, while *KRAS* mutations were also commonly found in tumors with a mucinous pattern. Other mutations were not associated with histologic subtypes (Supplemental Table 3).

Patients with tumors harboring *EGFR* and *BRAF* mutations were older than those with wild-type tumors, whereas *ALK* gene fusions and *ERBB2* mutations were associated with younger age at diagnosis (Supplemental Table 4). Driver gene mutation status was not associated with tumor stage.

## DISCUSSION

In this study, we evaluated the status of driver gene mutations in lung adenocarcinoma samples from 198 East Asian female never-smokers using the MassARRAY® LungCarta Panel and *ALK*, *ROS1*, and *RET* fusion assays. Approximately 79% of patients harbored driver gene mutations, and *EGFR* mutation (63%) was the most frequent driver mutation detected. Other genetic alterations occurred less frequently with *ALK* gene fusion, *KRAS* mutation, *ERBB2* mutation, *HER2* mutation, *ROS1* rearrangement, and *RET* rearrangement occurring in 7%, 4%, 1.5%, 1%, 1%, and 1% of cases, respectively. Most mutations were mutually exclusive.

Ethnic background is a well-established factor in NSCLC. Asian female non-smokers were the specific epidemiologic subgroup in this study, and a few previous large-scale studies have shown that Asian ethnicity is a prognostic factor of overall survival for NSCLC patients [15–17]. The link between ethnicity and cancer survival rates is likely due to differences in genetic background among ethnicities. Compared to Caucasians, East Asians with NSCLC have higher rates of *EGFR* mutations and lower rates of *KRAS* and *LKB1* mutations [18]. According to a recent meta-analysis of 94 studies, the rate of *EGFR* mutation in lung adenocarcinoma in Asians (47.9%) is higher than that in Westerners (19.2%), while *KRAS* (11.2%) and *LKB1* (4.0%) mutation rates are lower in Asians than in Westerners (26.1% and 16.2%, respectively) [19]. Smoking status is also an important factor in the development of lung adenocarcinoma. Lung cancers in never-smokers are more frequently associated with adenocarcinoma with *EGFR* mutations and less frequently with *KRAS* mutations [20]. In a recent study of lung adenocarcinomas, mutation rates for *EGFR* (39%), *KRAS* (4%)*, ALK* (15%), and *HER2* (5%) in never-smoker groups differed from rates in current or former smoker groups (10%, 35%, 4%, and 1%, respectively) [21]. Notably, the proportion of female lung cancers in never-smokers is much higher in East Asia than in Europe or the United States (60% vs 15–20%) [20].

Although many studies have investigated driver gene mutations in lung adenocarcinoma, only a few have focused specifically on Asian female never-smokers despite the demonstrated importance of this patient group for selection of targeted candidates for NSCLC therapy [22–24]. Zhang et al. analyzed *EGFR*, *KRAS*, *ALK*, *HER2*, and *BRAF* mutation in 349 Chinese never-smoker females with lung adenocarcinoma and discovered mutation rates of 76%, 5%, 4%, 2%, and 1% for *EGFR*, *HER2*, *ALK*, *KRAS*, and *HER2*, respectively, while only 12% of cases harbored no detected mutation [25]. In an analysis of mutations of 10 driver genes (*EGFR*, *KRAS*, *NRAS*, *HRAS*, *HER2*, *BRAF*, *ALK*, *PIK3CA*, *TP53*, and *LKB1*) in 52 lung adenocarcinomas from East Asian never-smokers including 41 women [26], Sun et al. found similar rates of genetic alterations: *EGFR* mutation in 79% of cases, *EML4-ALK* fusion in 6% of cases, *HER2* mutation in 4% of cases, and *KRAS* mutation in 2% of cases. In addition, only 10% of patients did not harbor any detected mutation in these genes. Ren et al. reported *EGFR* mutation in 70% and *ALK* rearrangement in 9.6% in adenocarcinomas from never-smoker Chinese women (see Supplemental Table 5 for summary of these studies) [27]. The incidences of mutation detected in this study are consistent with those of previous studies.

Several important features distinguish our study from previous studies. In our study, we analyzed a total of 26 known oncogenes and 3 fusion genes. We discovered that 1% of lung adenocarcinomas from East Asian never-smoker female patients harbored gene rearrangements of *ROS1* and *RET*, which have recently been recognized as driver genes in lung adenocarcinoma [28–30] but had not been evaluated in East Asian female never-smokers. We used a multiplex method that can be used in routine clinical practice and our results demonstrated that this method was comparable to traditional mutational analysis using polymerase chain reaction (PCR) for each gene. The use of a multiplexed PCR-based assay to genotype NSCLCs was recently demonstrated to be clinically feasible [31]. Moreover, we confirmed well-known associations of driver gene mutations with histologic subtypes and clinical characteristics. *EGFR* mutation correlated positively with older age and a predominantly acinar pattern [25, 32, 33], while *ALK* rearrangement was associated with younger age and solid histology [34, 35]. *KRAS* mutation was detected more frequently in mucinous adenocarcinoma [25, 36]. Furthermore, most *PIK3CA* mutations coexisted with other mutations [26]. The fact that our findings are consistent with those of previous studies supports the value of the multiplex method in mutation analysis and suggests that clinicopathologic associations are useful for determination of the priority of driver mutation tests.

The most outstanding result of our study was that driver gene mutations were detected in 79% of female never-smoker Asian patients. Targeted therapies for *EGFR* (erlotinib/gefitinib) and *ALK* (crizotinib) are currently available, and 70% of female never-smoker Asian patients with *EGFR* mutation (63%) or *ALK* rearrangement (7%) may benefit from such targeted therapies. An additional 10% of patients in our study may benefit from newly developed targeted drugs. A recent clinical trial described a partial response to the *EGFR/HER2* inhibitor BIBW2922 in a *HER2* mutant tumor [37], suggesting that this new drug is a promising treatment strategy. More comprehensive genomic analysis and deep sequencing may be necessary to identify genetic alterations in the remaining 20% of patients with no known mutations [26]. Notably, known driver gene mutations were found less frequently in mucinous adenocarcinoma in our study, suggesting the possibility of mutation in another driver gene in this histologic subtype.

In conclusion, lung cancer in never-smoker Asian females is a distinct entity, with the majority of these lung cancers developing from oncogenic mutations.

## METHODS

### Study population

Specimens were obtained from Samsung Medical Center (SMC) in Seoul, Korea with prior informed patient consent and approval by the Institutional Review Board of Samsung Medical Center. Between January 2008 and January 2013, 3796 consecutive patients underwent pulmonary resection with curative intent for primary lung cancer at our institute.

After excluding 1307 patients with non-adenocarcinoma (Figure 1A), the remaining 2489 patients consisted of 1302 men and 1187 women (Figure 1B). Of the 1187 female patients with lung adenocarcinoma, 422 patients were excluded due to a lack of smoking information. Of the remaining 765 female patients, 94.9% (726 women) were never-smokers (Figure 1C). Among the 726 never-smoker female pulmonary adenocarcinoma cases, 198 had sufficient tissue for genomic analysis and were included in this study.

### Data collection and histologic classification of adenocarcinoma

Study data were abstracted from in-hospital charts and electronic medical records by trained experienced nurses from the Departments of Medical Oncology, Surgical Oncology, Laboratory Medicine, Pathology, and Nursing at Samsung Medical Center. Baseline clinical characteristics included gender, age at diagnosis, smoking history, alcohol consumption status, date of diagnosis of advanced lung cancer, tumor histology, tumor stage, and ambulatory status at diagnostic work-up. Tumor stage was defined according to the seventh edition of the American Joint Committee on Cancer [38]. Smoking history and alcohol consumption status were determined by self-reported answers on questionnaires. Never-smokers were defined as patients who smoked less than 100 cigarettes over their lifetime. Histologic classification was determined according to the International Association for the Study of Lung Cancer, American Thoracic Society, and European Respiratory Society classification of lung adenocarcinomas [39]. All histologic patterns that covered at least 10% of the tumor area were recorded, and the predominant pattern was defined as the pattern that covered the largest portion of the tumor area.

### DNA and RNA extraction

Genomic DNA or RNA was extracted from lung tumors or distant histologically normal lung tissue using standard protocols (RNeasy Mini Kit and QIAamp DNA Mini Kit, Qiagen, Valencia, CA, USA).

### *ALK*, *ROS1*, and *RET* fusion assays

The nCounter™ gene expression assays were custom-designed and synthesized by NanoString Technologies (Seattle, WA, USA). Hybridization, sample cleanup, and digital reporter counts were performed according to the manufacturer's protocol. RNA was obtained from fresh-frozen tissues using the Qiagen RNeasy Mini Kit (Qiagen, Valencia, CA, USA). RNA concentration was assessed by spectrophotometry using the Nanodrop 8000 (Thermo-Scientific, Wilmington, DE, USA).

Samples were processed according to the gene expression protocol of NanoString Technologies. Briefly, total RNA was hybridized to a multiplexed mixture of custom-designed nCounter™ capture and reporter probes complementary to *ALK*, *ROS1*, and *RET* target sequences (Supplemental Table 2) for at least 16 h at 65°C. The samples were cleaned up and processed using an automated nCounter™ Sample Prep Station (NanoString Technologies). Unhybridized probes were removed, and the hybridization complex was immobilized onto a cartridge and aligned. Fluorescently labeled, color-coded reporters were subsequently imaged on an nCounter™ Digital Analyzer (NanoString Technologies) set at 1155 fields of view. Raw reporter counts were collected using nSolver software v1.0 (NanoString Technologies).

### LungCarta analysis

High-throughput multiplex mutation profiling was performed using the MassARRAY® LungCarta Panel Version 1.0 (Sequenom, San Diego, CA, USA). This panel permits screening of 214 mutations across 26 oncogenes and tumor suppressors with a limit of sensitivity of approximately 10% with the use of 480 ng DNA [17]. DNA was amplified using the OncoCarta PCR primer mix, unincorporated nucleotides were inactivated by shrimp alkaline phosphatase, and a single base extension reaction was performed using extension primers that hybridize adjacent to the mutation. Multiplexed reactions were spotted onto the SpectroChipII (Sequenom) using the MassARRAY Nanodispenser. Peaks with different mass were resolved by matrix-assisted laser desorption/ionization time-of-flight on the MassARRAY Compact Analyzer. A predefined ratio of expected normal allele to mutant allele at a specific nucleotide position allows mutations to be detected using primer extensions at that specific position. Because of the multiplexing capabilities of this assay, multiple mutations are detected simultaneously using one panel. Further details of the multiplex methodology can be found in the protocol provided by the manufacturer.

### Sanger sequencing

To validate the LungCarta analysis, we performed Sanger sequencing of *EGFR* in 11 selected cases (4 cases of *EGFR* E746_A750del, 4 cases of *EGFR* L858R, and 3 cases of *KRAS* G12D) according to the method described previously by our group [40].

### Statistical analysis

Pearson's chi-squared, Fisher's exact, and independent *t* tests were used as indicated. All tests were two sided, and a *p*-value of < 0.05 was considered statistically significant. Statistical analyses were performed using SPSS software (SPSS Inc., Chicago, IL, USA).

## SUPPLEMENTAL TABLES




