# Supplementary material for: Lung cancer in never-smoker Asian females is driven by oncogenic mutations, most often involving EGFR
Source: Oncotarget. 2015 Feb 28;6(7):5465–74. doi: 10.18632/oncotarget.2925 (PMC4467161; doi:10.18632/oncotarget.2925)
Supplement: Supplementary file 1 [file oncotarget-06-5465-s001.pdf]

SUPPLEMENTAL TABLES

Supplemental Table 1: List of mutations in all patients

ID	Mutation
1	-
2	-
3	E746_A750del in EGFR, H1047R in PIK3CA
4	-
5	L858R in EGFR
6	-
7	-
8	ALK fusion
9	-
10	E746_A750del in EGFR
11	L858R in EGFR
12	L858R in EGFR
13	-
14	-
15	V769_D770insASV in EGFR
16	L858R in EGFR
17	Q61R in NRAS
18	E746_A750del in EGFR
19	E746_A750del in EGFR
20	E746_A750del in EGFR
21	E746_A750del in EGFR
22	-
23	L858R in EGFR
24	E746_A750del in EGFR, E542K in PIK3CA
25	L858R in EGFR
26	H773_V774insPH in EGFR
27	L747_A750 > P in EGFR
28	L858R in EGFR
29	E746_A750del in EGFR
30	ALK fusion
31	L858R in EGFR
32	L858R in EGFR, R282W in TP53

(*Continued*)

ID	Mutation
33	G12V in KRAS
34	L747–752del in EGFR
35	P753S,L747S,L747_P753 > S in EGFR
36	P753S,L747S,L747_P753 > S in EGFR
37	E746_A750del in EGFR
38	E746_A750del in EGFR
39	Q61H in KRAS
40	G12C in KRAS
41	L858R in EGFR
42	-
43	L858R in EGFR
44	ALK fusion
45	E746_A750del in EGFR
46	L858R in EGFR
47	L858R in EGFR
48	RET fusion
49	E746_A750del in EGFR
50	M774_A775insAYVM in ERBB2
51	-
52	P753S,L747S,L747_P753 > S in EGFR
53	L858R in EGFR
54	E709KH, L858R in EGFR
55	E545K in PIK3CA
56	ALK fusion
57	L858R in EGFR
58	L747_S752del, L747_A750 > P in EGFR
59	L858R in EGFR
60	L858R in EGFR
61	ALK fusion
62	ALK fusion
63	L858R in EGFR
64	E746_A750del in EGFR, H1047L in PIK3CA
65	E746_A750del in EGFR
66	-
67	-

(Continued)

ID	Mutation
68	E746_A750del in EGFR
69	-
70	L858R in EGFR
71	ALK fusion
72	V600E in b RAF
73	-
74	V769_D770insASV in EGFR
75	ALK fusion
76	E746_A750del in EGFR
77	E746_A750del in EGFR
78	L858R in EGFR
79	L858R in EGFR, R248Q in TP53
80	-
81	L747_A750 > P in EGFR
82	E746_A750del in EGFR
83	-
84	-
85	-
86	L858R in EGFR
87	L858R in EGFR
88	L747_T751del in EGFR
89	V600E in b RAF
90	L858R, T790M in EGFR
91	L747_A750 > P in EGFR
92	L858R in EGFR
93	RET fusion
94	L858R in EGFR
95	L858R in EGFR
96	V769_D770insASV in EGFR
97	-
98	L858R in EGFR
99	-
100	L858R in EGFR
101	E746_E749del/I744_K745insKIPVAI/E746_A750 > IP in EGFR
102	L858R in EGFR, R175H in TP53

(*Continued*)

ID	Mutation
103	E746_A750del in EGFR
104	L858R in EGFR
105	E746_E749del/I744_K745insKIPVAI/E746_A750 > IP in EGFR
106	-
107	-
108	E709AGV,L858R in EGFR
109	L747_A750 > P in EGFR
110	-
111	ALK fusion
112	L858R in EGFR
113	-
114	L858R in EGFR
115	L858R in EGFR
116	L858R in EGFR
117	-
118	E746_A750del in EGFR
119	E746_A750del in EGFR
120	-
121	L858R in EGFR, R175H in TP53
122	-
123	S752F,E746_S752 > V in EGFR
124	L858R in EGFR
125	E746_A750del in EGFR
126	E746_A750del in EGFR
127	L858R in EGFR
128	L858R in EGFR
129	-
130	M774_A775insAYVM in ERBB2
131	T751P,L747_A750 > P,L747_T751 > P in EGFR
132	G719S, S768I in EGFR
133	L858R in EGFR
134	G12D in KRAS
135	-
136	ALK fusion
137	L858R in EGFR

(*Continued*)

ID	Mutation
138	-
139	P753S,L747S,L747_P753 > S in EGFR
140	H773_V774insPH in EGFR
141	-
142	L858R in EGFR
143	L747_A750 > P in EGFR
144	L858R in EGFR
145	-
146	-
147	L858R in EGFR
148	L858R in EGFR
149	E746_A750del in EGFR
150	L858R in EGFR
151	ALK fusion
152	L858R in EGFR
153	E746_A750del, T790M in EGFR
154	-
155	L858R in EGFR
156	L858R in EGFR
157	-
158	L858R in EGFR
159	L858R in EGFR
160	L858R in EGFR
161	L858R in EGFR
162	L858R in EGFR
163	L858R in EGFR
164	-
165	L861Q in EGFR
166	ALK fusion
167	L858R in EGFR
168	-
169	L858R in EGFR
170	L747_T751del in EGFR
171	L858R in EGFR
172	H773_V774insH in EGFR

(*Continued*)

ID	Mutation
173	E746_A750del in EGFR
174	V769_D770insASV in EGFR
175	E746_A750del in EGFR
176	L861Q in EGFR
177	E746_A750del in EGFR
178	-
179	G12D in KRAS
180	ALK fusion
181	ALK fusion
182	E746_A750del in EGFR
183	L858R in EGFR
184	ROS fusion
185	G12D in KRAS, A775_G776insYVMA in ERBB2, E542K in PIK3CA
186	T751P,L747_A750 > P,L747_T751 > P in EGFR
187	E746_A750del in EGFR
188	L858R in EGFR
189	E746_A750del in EGFR
190	E746_A750del in EGFR
191	ROS fusion
192	E746_A750del in EGFR
193	G12C in KRAS
194	G12D in KRAS
195	E746_A750del, E746K/E746_T751 > IP in EGFR
196	-
197	-
198	-

Supplemental Table 2: Validation of gene mutations by sanger sequencing

ID	LungCarta	Direct sequencing
3	EGFR E746_A750del	EGFR E746_A750del
10	EGFR E746_A750del	EGFR E746_A750del
11	EGFR L858R	EGFR L858R
12	EGFR L858R	EGFR L858R
16	EGFR L858R	EGFR L858R
19	EGFR E746_A750del	EGFR E746_A750del
20	EGFR E746_A750del	EGFR E746_A750del
23	EGFR L858R	EGFR L858R
134	KRAS G12D	KRAS G12D
179	KRAS G12D	KRAS G12D
185	KRAS G12D	KRAS G12D

Supplemental Table 3: Relationship between driver mutation status and histologic pattern

Supplemental Table 4: Association of driver gene mutation and patient age

Driver gene	Status	Age mean ± sd	*p value*
EGFR	**mutated**	61.3 ± 9.3	0.042
	**wild**	58.3 ± 11.0	
ALK	**mutated**	51.3 ± 11.6	0.004
	**wild**	60.8 ± 9.6	
KRAS	**mutated**	61.0 ± 8.0	0.872
	**wild**	60.1 ± 10.1	
PIK3CA	**mutated**	59.6 ± 7.6	0.809
	**wild**	60.2 ± 10.1	
TP53	**mutated**	57.8 ± 11.1	0.64
	**wild**	60.2 ± 10.0	
ERBB2	**mutated**	47.0 ± 10.8	0.044
	**wild**	60.4 ± 9.9	
BRAF	**mutated**	75.5 ± 3.5	0.031
	**wild**	60.0 ± 10.0	
ROS1	**mutated**	61.0 ± 9.9	0.946
	**wild**	60.2 ± 10.1	
RET	**mutated**	57.0 ± 9.9	0.581
	**wild**	60.2 ± 10.1	

Supplemental Table 5: Summary of previous studies investigating driver gene mutation in lung adenocarcinoma of East Asian non-smokers

Author	Year	Country	Number of patients	Female ratio	*EGFR*	*ALK*	*KRAS*	*ERBB2*	*BRAF*	*ROS1*	*RET*	Unknown
Sun et al.[1]	2010	China	52	78.80%	78.8%	5.8%	1.9%	3.8%	NA	NA	NA	9.6%
Ren et al.	2012	China	104	100%	70.2%	9.6%	NA	NA	NA	NA	NA	20.2%
Zhang et al.	2012	China	349	100%	76.2%	4.3%	2.0%	4.6%	0.6%	NA	NA	12.3%
Kim et al.	2014	Korea	162	84.60%	50.6%	8.6%	3.7%	NA	NA	3.1%	NA	33.9%
Li et al.	2014	China	202	78.70%	75.3%	5.0%	2.0%	6.0%	0.0%	1.0%	NA	12.9%
Present study	2014	Korea	198	100%	62.6%	7.1%	4.0%	1.5%	1.0%	1.0%	1.0%	20.7%

NA, Not applicable
